# Validation of a New Histopathologic Risk Model in Early Oral Tongue Cancer: A Combination of a Modified Worst Pattern of Invasion and a New Tumor Budding Score

**DOI:** 10.1097/PAS.0000000000002433

**Published:** 2025-06-11

**Authors:** Alhadi Almangush, Tuula Salo, Caj Haglund, Luiz Paulo Kowalski, Jaana Hagström, Ricardo D. Coletta, Antti A. Mäkitie, Ilmo Leivo

**Affiliations:** * Department of Pathology; ‡ Research Program in Systems Oncology, Faculty of Medicine; ‖ Department of Oral and Maxillofacial Diseases; ¶ Research Programs Unit, Translational Cancer Medicine; Departments of # Surgery, University of Helsinki; §§ Otorhinolaryngology—Head and Neck Surgery, University of Helsinki and Helsinki University Hospital, Helsinki; † Institute of Biomedicine, Pathology, University of Turku, Turku; †† Department of Oral Pathology and Radiology, University of Turku; ¶¶ Turku University Central Hospital, Turku, Finland; § Libyan Authority for Scientific Research, Tripoli, Libya; ** Department of Head and Neck Surgery and Otorhinolaryngology, A.C. Camargo Cancer Center, and University of Sao Paulo Medical School, Department of Head and Neck Surgery; ‡‡ Department of Oral Diagnosis and Graduate Program in Oral Biology, School of Dentistry, University of Campinas, Piracicaba, São Paulo, Brazil; ‖‖ Division of Ear, Nose and Throat Diseases, Department of Clinical Sciences, Intervention and Technology, Karolinska Institutet and Karolinska University Hospital, Stockholm, Sweden

**Keywords:** early stage, oral tongue cancer, prognosis, modified worst pattern of invasion, new tumor budding score, tumor cell dissociation

## Abstract

Oral tongue squamous cell carcinoma (OTSCC) is the most common cancer of the oral cavity. A new histopathologic risk assessment has been recently introduced and we sought to validate its prognostic value in a large multicenter cohort of early-stage OTSCC. A total of 310 cases treated for early-stage OTSCC were included in this study. The assessment of modified worst pattern of invasion and a recently developed tumor budding score were performed in hematoxylin and eosin-stained sections. A statistically significant association was observed in the multivariable analysis between high score of the new risk model and worse disease-specific survival (HR: 2.54, 95% CI: 1.48-4.37, *P*<0.001). Similarly, in disease-free survival, the high-risk group was significantly associated with poor survival (HR: 1.66, 95% CI: 1.07-2.58, *P*=0.024). In conclusion, the new histopathologic risk model is a powerful prognostic indicator and can be assessed as part of routine diagnostic practice. Early-stage OTSCC patients with a high-risk score have a poor prognosis and therefore require a multimodality treatment strategy with a close clinical follow-up.

Oral tongue squamous cell carcinoma (OTSCC) is a common malignancy of the oral cavity and its incidence is increasing in many countries.^1–3^ In addition to the effects of typical risk factors such as smoking, the incidence of oral cavity cancer is also rising in nonsmokers.^4^ In early-stage OTSCC, there are dramatic variations in treatment response ranging from favorable outcomes to about a 20% rate of recurrence, occurrence of metastasis and/or cancer-related mortality.^5^ Thus, predicting the behavior of OTSCC accurately, especially in early-stage cases remains crucial for developing an optimal treatment strategy.

The tumor invasive front (IF) has been studied for decades and it is well-documented that some critical events, such as epithelial to mesenchymal transition (EMT), occur at the IF area.^6,7^ Therefore, the histopathologic characteristics of the IF area can aid in predicting clinical scenarios accurately. Tumor cell dissociation and active invasion typically occur at the IF area and they signify greatly increased movement of tumor cells including both local infiltration and metastasis.^8^ Notably, the prognostic significance of tumor cell dissociation is more important than that of tumor differentiation in early OTSCC.^8–10^ To assess tumor cell dissociation, the worst pattern of invasion (WPOI) was initially described in 5 categories,^11^ and recently, the modified WPOI has been simplified into 3 categories.^12^

Tumor budding is an emerging histomorphologic prognostic marker defined as the presence of small clusters of fewer than 5 cancer cells at the invasive front area.^13^ In studies across different tumor types including OTSCC, tumor budding has been correlated with aggressive clinical behavior including recurrence, metastasis, and poor survival.^13,14^ Importantly, tumor budding has also correlated with EMT, with the buds playing a key role in tumor-host interactions.^13^ The incorporation of a new tumor budding score with modified WPOI criteria has been recently introduced by Chang et al.^12^ The new tumor budding score combines tumor budding density and tumor cell dissociation. The aim of the current study is to assess the prognostic value of the new histopathologic risk model in early-stage OTSCC.

## MATERIALS AND METHODS

We included 310 patients treated for early-stage (cT1-T2N0M0) OTSCC, who were also studied in our recent research.^15^ These patients were treated at 1 of the 5 Finnish university hospitals (Helsinki, Tampere, Turku, Kuopio, Oulu) or at the A.C. Camargo Cancer Center, São Paulo, Brazil. Ethical permission was obtained from the above-listed university hospitals, the Finnish National Supervisory Authority for Welfare and Health, and the Brazilian Human Research Ethics Committee. We used the World Health Organization (WHO) criteria to determine tumor grade.^16^

**FIGURE 1:**
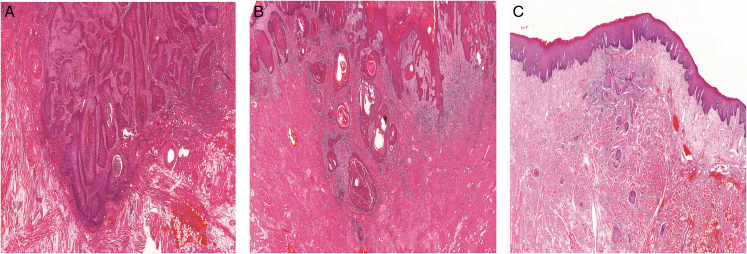
Modified worst pattern of invasion type 1 (A), type 2 (B) and type 3 (C). Hematoxylin and eosin staining.

We evaluated the modified WPOI following the recently introduced criteria,^12^ which simplify classification from the 5 categories of the original WPOI into 3 categories (Fig. 1) and eliminate the counting of cancer cells from the assessment. Thus, the modified WPOI is described as follows: In modified WPOI-1, cancer cell nests are confined within the main boundary of the tumor. This feature differs from modified WPOI-2 and modified WPOI-3, in which some of cancer nests extend beyond the main tumor boundary. Then, the distance between tumor satellites will differentiates modified WPOI-2 from modified WPOI-3. Modified WPOI-2 includes tumors with a distance of <1 mm between the tumor nests. Modified WPOI-3 includes tumors with a distance of 1 mm or more from the main tumor mass or the nearest tumor satellite. The modified WPOI assigns 0 point (for modified WPOI-1), 1 point (for modified WPOI-2), and 2 points (for modified WPOI-3).

For the evaluation of tumor budding, cell clusters containing fewer than 5 cancer cells at the invasive front area were counted, and single cancer cells were also scored.^12^ The new tumor budding score, combining tumor budding density and cell dissociation was defined as follows:

0: No single tumor cells and no tumor buds present.

1: Only low-density tumor budding (fewer than 5 buds present).

**FIGURE 2:**
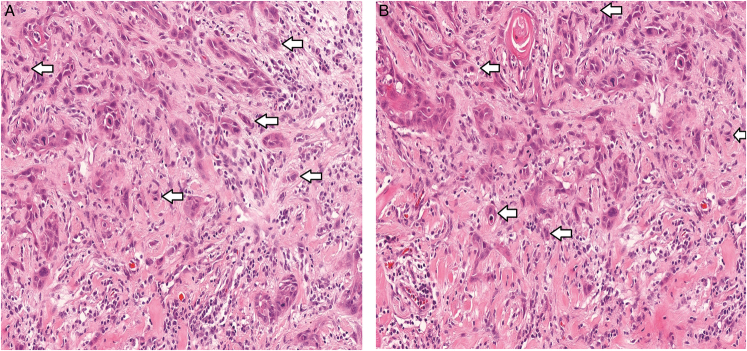
High tumor budding score (A and B). Hematoxylin and eosin staining.

2: Single tumor cells and/or high-density tumor budding (5 buds or more) present (Fig. 2).

Histopathologic assessments of the included parameters were performed on resection samples. Two observers (A.A. and I.L.) arranged a training scoring session to familiarize themselves with the evaluation of the new risk model. The model, as designed in Chang et al,^12^ study by combining the modified WPOI and the new tumor budding score, and the cases were categorized into 3 groups: low risk (score 0 to 1), intermediate risk (score 2 to 3), and high risk (score 4). Cases with discordant scores were addressed by a discussion in an interactive joint review session with both observers until an agreement about the score was reached.

### Statistical Method

All statistical analyses were performed using SPSS 27. A hazard ratio (HR) and 95% CI were reported for both univariable and multivariable analyses. Kaplan-Meier survival curves were produced to report the relationships between the risk groups. *P*-value of <0.05 was considered statistically significant.

## RESULTS

In this cohort, there were 164 men (52.9%) and 146 women (47.1%) with a median age of 62 years, and a median follow-up time of 57 months. During the follow-up, 63 patients (20.3%) died due to OTSCC, 95 patients (30.6%) died of other causes, and 152 patients (49.0%) were alive. Regarding tumor staging, 123 cases (39.7%) were classified as T1N0M0 and 187 (60.3%) were T2N0M0. One hundred five tumors (33.9%) were well differentiated, 130 tumors (41.9%) were moderately differentiated, and 75 tumors (24.2%) were poorly differentiated. In assessment of the modified histopathologic risk model, a moderate to good agreement (Kappa=0.73) was found between the observers.

Points were assigned according to the modified WPOI, so that 100 (32.3%) cases were assigned 0 points (ie, type 1), 167 (53.9%) were assigned 1 point (ie, type 2), and 43 (13.9%) were assigned 2 points (ie, type 3). With regard to the tumor budding score, there were 90 (29.0%) cases assigned 0 points, 55 (17.7%) cases assigned 1 point, and 165 (53.2%) cases assigned 2 points. This resulted in a risk score combing the modified WPOI and tumor budding, so that 61 (19.7%) cases received 0 point, 38 (12.3%) cases received 1 point, 62 (20.0%) cases received 2 points, 120 (38.7%) cases received 3 points, and 29 (9.4%) cases received 4 points. There was a statistically significant association between high-risk cases and the presence of perineural invasion (*P*<0.001). No significant association (*P* > 0.05) was found between the new risk model and any of the other clinicopathologic features (Table 1).

**TABLE 1:** Relationship Between the New Histopathologic Risk Model and Clinicopathologic Characteristics of Early-Stage Oral Tongue Cancer

	New risk model		
	Low risk	High risk	
Variable	Number, n (%) 161 (51.9%)	Number, n (%) 149 (48.1%)	*P*
Age			0.65
≤60 y	65 (50.4)	64 (49.6)	
>60 y	96 (53.0)	85 (47.0)	
Sex			0.59
Men	88 (53.3)	77 (46.7)	
Women	73 (50.4)	72 (49.7)	
TNM stage			0.29
T1N0M0	69 (55.6)	55 (44.4)	
T2N0M0	92 (49.5)	94 (50.5)	
Perineural invasion			0.001
None	149 (55.6)	119 (44.4)	
Present	12 (28.6)	30 (71.4)	
WHO grade			0.07
Well differentiated	60 (57.1)	45 (42.9)	
Moderately differentiated	71 (54.2)	60 (45.8)	
Poorly differentiated	30 (40.5)	44 (59.5)	

**FIGURE 3:**
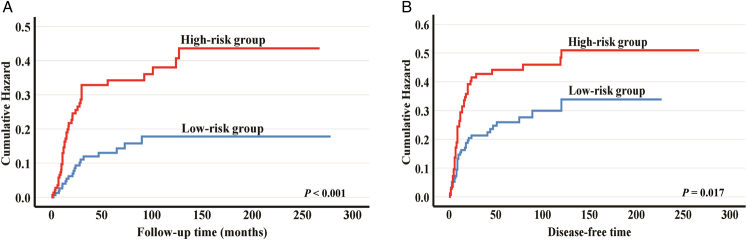
Kaplan-Meier survival curves presenting a significantly poor prognosis for cases having high-risk score as categorized by the new histopathologic risk model. A, Disease-specific survival. B, Disease-free survival.

In predicting cancer-related mortality (ie, disease-specific survival), a worse survival was observed in both the intermediate-risk group (HR: 2.25, 95% CI: 1.14-4.42, *P*=0.019) and the high-risk group (HR: 2.98, 95% CI: 1.19-7.41, *P*=0.019). Thus, we recategorized the cases into 2 risk groups with points 0, 1, and 2 as low-risk and cases with points 3 and 4 as high risk (Table 2). The HR for this high-risk group was 2.47 (95% CI: 1.45-4.19, *P*<0.001) in univariable analysis and 2.54 (95% CI: 1.48-4.37, *P*<0.001) in multivariable analysis. For disease-free survival, the high-risk group of the new risk model was also associated with a worse survival (HR: 1.67, 95% CI: 1.09-2.55, *P*=0.019) in both univariable and multivariable analysis (HR: 1.66, 95% CI: 1.07-2.58, *P*=0.024). Using Kaplan-Meier survival curves (Fig. 3), high-risk score cases presented a significantly worse prognosis.

**TABLE 2:** Disease-Specific Survival and Disease-Free Survival Analyses of the Prognostic Significance of the New Histopathologic Risk Model and Clinicopathologic Features of 310 Cases of Early-Stage Oral Tongue Cancer

	Disease-specific survival		Disease-free survival	
	Univariable analysis	Multivariable analysis	Univariable analysis	Multivariable analysis
Parameter	HR (95%CI), *P*	HR (95%CI), *P*	HR (95%CI), *P*	HR (95%CI), *P*
Age				
≤60 y	Reference	Reference	Reference	Reference
>60 y	1.88 (1.10-3.19), *P*=0.021	2.19 (1.26-3.79), *P*=0.005	1.76 (1.13-2.76), *P*=0.01	1.89 (1.19-3.00), *P*=0.007
Sex				
Men	Reference	Reference	Reference	Reference
Women	1.22 (0.75-2.00), *P*=0.43	1.19 (0.71-1.98), *P*=0.51	1.06 (0.69-1.62), *P*=0.79	0.92 (0.59-1.43), *P*=0.71
Stage				
T1N0M0	Reference	Reference	Reference	Reference
T2N0M0	1.49 (0.87-2.55), *P*=0.15	1.67 (0.96-2.90), *P*=0.07	0.86 (0.56-1.31), *P*=0.48	0.84 (0.54-1.31), *P*=0.44
Perineural invasion				
None	Reference	Reference	Reference	Reference
Present	1.27 (0.64-2.49), *P*=0.50	0.91 (0.46-1.83), *P*=0.79	1.48 (0.85-2.58), *P*=0.17	1.33 (0.75-2.38), *P*=0.33
WHO grade				
Well differentiated	Reference	Reference	Reference	Reference
Moderately differentiated	1.68 (0.92-3.07), *P*=0.09	1.92 (1.05-3.54), *P*=0.04	1.11 (0.68-1.82), *P*=0.67	1.18 (0.72-1.93), *P*=0.52
Poorly differentiated	1.60 (0.80-3.20), *P*=0.18	1.81 (0.89-3.69), *P*=0.10	1.20 (0.69-2.10), *P*=0.52	1.34 (0.76-2.38), *P*=0.32
New Risk model				
Low risk (score 0-2)	Reference	Reference	Reference	Reference
High risk (score 3-4)	2.47 (1.45-4.19), *P*<0.001	2.54 (1.48-4.37), *P*<0.001	1.67 (1.09-2.55), *P*=0.019	1.66 (1.07-2.58), *P*=0.024

Among the routinely reported histopathologic parameters, the depth of invasion has shown a significant prognostic value in predicting patients’ survival, both in univariable (HR: 3.03, 95% CI: 1.79-5.11, *P*<0.001) and in multivariable analysis (HR: 3.15, 95% CI: 1.83-5.42, *P*<0.001).

## DISCUSSION

Assessing the clinical behavior of early-stage OTSCC is sometimes challenging due to worse survival of many cases. In such instances, the choice of elective neck dissection versus. wait-and-see policy is the most challenging consideration. The significance of cellular dissociation from the main tumor mass has been emphasized in recent research in oral tongue cancer^8,10^ and other cancers as well.^13^ In this study, we found that the new histopathologic risk model,^12^ which combines the modified worst pattern of invasion and the new tumor budding score, is a powerful prognostic indicator for early-stage OTSCC. Traditionally, OTSCC has been histopathologically graded according to the WHO classification into well, moderately, and poorly differentiated tumors.^16^ However, this grading system does not provide a good risk stratification, especially in early-stage OTSCC.^9,17^ Recent research has emphasized the clinical significance of assessing parameters relevant to cellular dissociation, namely, tumor budding and the pattern of tumor invasion.^8^ Of note, the assessment of cellular dissociation has also been successfully conducted in preoperative samples.^18^ Furthermore, a combination of tumor dissociation parameters has been recently reported with valuable results for risk stratification.^10^ Most recently, Chang et al^12^ proposed a new histopathologic risk model based on the assessment of modified WPOI and a new tumor budding score. As presented in Table 2, this newly proposed risk model has a promising prognostic value superior to the WHO grading system.

In addition to its prognostic significance, the modified WPOI, compared with the original WPOI, has ideally simplified the evaluation as it focuses on the assessment of the tumor border, and the need for cell counting has been eliminated.^12^ This reduces the subjectivity in the assessment. The findings of our current study are supported by evidence from previous research where histologic characteristics related to tumor cell dissociation correlated significantly with survival in oral cancer,^19^ in other subsites of head and neck cancer^20^ and in upper aerodigestive tract.^21^ Specifically, tumor budding is a recognizable histopathologic manifestation of cancer cell invasion and metastasis.^22^ In many previous studies, tumor budding has been reported as a reliable prognostic biomarker in different subsites of oral cavity cancer, including the oral tongue, and this has been confirmed in recent meta-analyses.^14,23^ In addition, interobserver reproducibility in the assessment of tumor budding has been widely documented as good to perfect.^24–26^ At the molecular level, cells in tumor buds usually show dysregulation of the expression of E‑cadherin, vimentin and β‑catenin.^13,24,27^ In addition, it has also been proposed that budding cells could be the origin of circulating cancer cells.^28^ Thus, the combination of these 2 dissociative features is clinically relevant for predicting tumor aggressiveness.

Lymph node involvement has been widely reported as an adverse prognostic indicator in patients with oral tongue cancer.^29^ Of note, Chang et al^12^ have shown that their modified histopathologic risk model has a significant prognostic value in predicting lymph node metastasis. Furthermore, tumor budding, one of the parameters that constitute the model, has been widely associated with lymph node metastasis in many recent reports on different types of cancer including oral cancer.^13^ In our current study, however, a limitation was that the relationship between the proposed model and the pathologic status of the lymph nodes (ie, pN) was not analyzed, as the inclusion criteria in this multicenter study were to include early-stage cases with clinically negative neck (ie, cT1-T2N0). Therefore, pathologic staging was not provided by the participating hospitals. Other limitations of this study include the retrospective nature of our cohort. In addition, data for some prognostic factors including margin status, p53 score and HPV status were not available in many patients and therefore we could not include them in our survival analysis. Even with these limitations, our analysis has confirmed the usefulness of the newly introduced histopathologic risk model in predicting the patient’s survival in a large multicenter cohort of early-stage OTSCC. The discohesive tumor cells have an enhanced propensity for invasion and metastasis.^12^ Therefore, the validated risk model, which uses tumor discohesion parameters (tumor budding and modified WPOI), can aid in identifying those cases with a high risk of poor survival. This carries the potential to aid in selecting the best treatment approach, including multimodality treatments in cases diagnosed at an early stage.
